# Prevalence of sarcopenia in idiopathic dropped head syndrome patients is similar to healthy volunteers

**DOI:** 10.1038/s41598-021-95031-5

**Published:** 2021-08-10

**Authors:** Tatsuya Igawa, Ken Ishii, Norihiro Isogai, Akifumi Suzuki, Masahiro Ishizaka, Haruki Funao

**Affiliations:** 1 Department of Orthopaedic Surgery, School of Medicine, International University of Health and Welfare, 852 Hatakeda, Narita City, Chiba 286-8520 Japan; 2 Department of Orthopaedic Surgery, School of Medicine, International University of Health and Welfare Narita Hospital, 852 Hatakeda, Narita City, Chiba 286-8520 Japan; 3 Department of Orthopaedic Surgery and Spine and Spinal Cord Center, International University of Health and Welfare Mita Hospital, 1-4-3, Mita, Minato-ku, Tokyo, 108-8329 Japan; 4 Department of Rehabilitation, International University of Health and Welfare Mita Hospital, 1-4-3, Mita, Minato-ku, Tokyo, 108-8329 Japan; 5 Department of Physical Therapy, School of Health Science, International University of Health and Welfare, 2600-1, Kitakanemaru, Ohtawara, Tochigi 323-8501 Japan

**Keywords:** Neurology, Neurological disorders, Neuromuscular disease

## Abstract

Dropped head syndrome (DHS) exhibits cervical deformity due to weakness of the cervical extensor group, and sarcopenia is characterized by progressive and systemic reduction in skeletal muscle mass. These clinical finding are associated with reduced activity of daily living, reduced quality of life, and increased risk of mortality. We collected and reviewed prospective registry data for 16 patients with idiopathic DHS continuously collected without dropping out and 32 healthy individuals who matched their gender and age. The prevalence of sarcopenia and body composition data were compared. There were no differences in the prevalence of sarcopenia, appendicular muscle mass, and leg muscle mass between DHS patients and the healthy elderly. Trunk muscle mass in DHS patients was significantly lower than that in healthy individuals. A significant correlation was found between appendicular muscle mass and trunk muscle mass in healthy subjects but not in DHS patients. Sarcopenia was not associated with the onset of idiopathic DHS. The prevalence of sarcopenia was not high in patients with idiopathic DHS due to the preservation of their appendicular skeletal muscle mass. Patients with DHS were characterized by a significant loss of trunk muscle mass that may be related to the disease but not aging.

## Introduction

DHS is a rare condition in which weakness of the cervical extensor muscles causes horizontal gaze disorder, gait disturbance, and dysphagia^1^. While muscle atrophy also develops in neuromuscular and muscular disorders, idiopathic DHS caused by an unexplained cervical extensor weakness is a problem for many older patients^1–4^. With the aging of society, the prevalence of the disease may increase further. Although there are some reports on muscle degeneration in DHS, many aspects of the disease are still unknown^1,2,5,6^. Sarcopenia, an age-related loss of skeletal muscle and strength, is widely accepted as a geriatric disease that can exacerbate motor dysfunction and increase the risk of adverse consequences such as falls and disabilities in patients with DHS. Sarcopenia, a term proposed by Rosenberg in 1989^7,8^, was initially focused solely on skeletal muscle mass. The disease now includes muscle strength and/or physical dysfunction in addition to the loss of skeletal muscle mass that is often seen in old age^9–12^. The clinical finding of sarcopenia is associated with reduced activity and quality of life and increased risk of death^13^. Akune et al.^14^ reported that its prevalence, based on the Asia Working Group for Sarcopenia (AWGS) consensus^15^, was 13.8% for older men and 12.4% for women. There are very few reports on the prevalence of sarcopenia in patients with DHS^16,17^. As the muscle strength and physical function were not evaluated in these studies, the prevalence may be overestimated. Lin et al.^18^ used electromyographic analysis to demonstrate abnormal muscle activities in the neck and trunk muscles such as the trapezius, levator scapulae, and sternocleidomastoid muscles in patients with DHS. However, the relationship between DHS and muscle mass, including the trunk muscles, is unknown. The prevalence of sarcopenia can exacerbate the disability of DHS patients, and the investigation of muscle mass and physical function are important for understanding the pathophysiology of these patients.

Therefore, we aimed to clarify (1) the prevalence of sarcopenia in patients with DHS, including muscle strength and physical performance in accordance to the revised 2019 AWGS standard^15^ and (2) the relationship between the decrease in skeletal muscle mass between the limbs and trunk of DHS patients.

## Materials and methods

This study was approved by the Institutional Review Board of International University of Health and Welfare (IRB#5-17-7, 5-19-20, 18-Io-158-2). All participants provided written informed consent. All procedures conducted in this study were in accordance with the 1964 Helsinki declaration and its later amendments or comparable ethical standards.

### Participants

**Figure 1:**
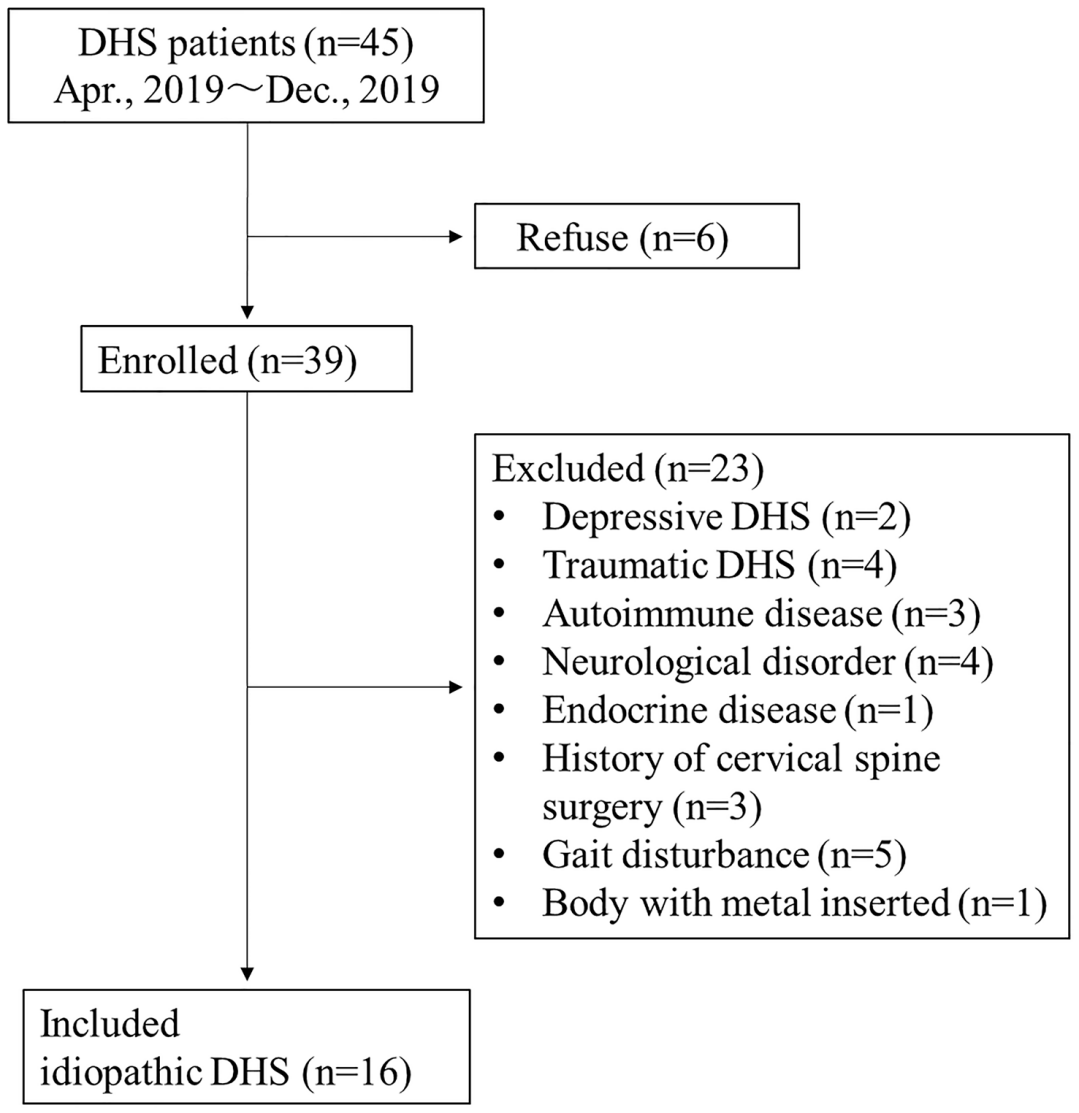
Flowchart of patient enrollment. *DHS* dropped head syndrome.

This cross-sectional study examined DHS patients and healthy volunteers living in the community at a single institution in the Kanto region of Japan. The subjects were female patients with idiopathic DHS were continuously collected from April to December 2019. The diagnostic criteria for DHS was defined as the presence of weakness of the cervical extensor muscles and difficulty in horizontal gaze in the standing position. Those with depression and post-traumatic DHS, as well as those with autoimmune disease, neurological disease, endocrine disease, history of cervical spine surgery, malignant tumor, severe organ failure, gait disturbance, and metal implanted in the body were excluded. Gait disturbance was defined as having difficulty walking 10 m independently. Sixteen DHS patients who met the eligibility criteria were included in this study (mean age 75.4 years, range 64–88) (Fig. 1). From January 2019 to February 2020, 32 of the 96 age and gender-matched healthy volunteers (27 males and 69 females, mean age 76.4 years, range 60–94) living in Kanto region of Japan were selected as control groups. Healthy subjects were recruited by the public relations department of the city and voluntarily participated in a care prevention project sponsored by the city. Each participant signed a written consent form. The study complied with the declaration of Helsinki and was approved by the Ethics Review Committee (approval number: 5-17-7, 5-19-20, 18-Io-158-2).

### Data collection

The body composition, handgrip strength, and walking speed were measured before the exercise and at least 3 h after eating. Body composition was measured by multifrequency bioelectrical impedance analysis (BIA) using a body composition analyzer (MC780, Tanita Inc., Tokyo, Japan). Handgrip strength was measured by gripping the digital grip dynamometer with maximum effort (T.K.K.5401, Takei Scientific Instrument, Niigata, Japan). The measurement was performed twice on each side, and the maximum value among them was used as the representative value^19^. Walking speed was measured once at the participant’s usual speed^20^. Measurements were also obtained for body mass, body mass index, body fat mass, lean body mass, arm muscle mass, leg muscle mass, and trunk muscle mass. The skeletal muscle mass index (SMI) was then calculated by dividing the appendicular skeletal muscle mass by the square of the participant’s height. Sarcopenia was assessed based on the criteria of the AWGS 2019 algorithm^15^. Participants who met the criteria of both low handgrip strength and/or low gait speed and low muscle mass were considered to have sarcopenia^15^. The fat mass index (FMI) and fat-free mass index (FFMI) were calculated by dividing the body fat mass and lean body mass by the square of the participant’s height, respectively. In addition, the FFMI to FMI (FFMI/FMI) ratio was measured to eliminate the influence of physique and to investigate the relationship between lean body mass and fat mass.

### Statistical analysis

In this study, the subjects to be analyzed in the control group were selected using propensity score matching. To estimate the propensity score, we fitted a logistic regression model for gender and age with caliper width set to 0.05 times the standard deviation of the logit of the propensity score. One-to-two matching without replacement was completed using the nearest neighbor match on the logit of the propensity score. Power analysis was performed using G*Power 3.1 (Heinrich Heine University, Düsseldorf, Germany). The sample size (16 patients and 32 controls) was determined to be able to detect a difference between both groups, assuming an effect size of 0.94, a type I error probability of 5%, and a type II error probability of 15% (i.e. power of 85%). Effect size was determined based on the result of the SMI value of a previous study^17^. The clinical characteristics and the other measurements were not normally distributed according to the Kolmogorov–Smirnov test; therefore, a parametric statistical analysis was performed. Student's t-test was used for the data to compare the values of the two groups. Diagnosis of sarcopenia was compared by Fisher’s exact test. Pearson correlations were calculated between the SMI and trunk muscle mass and the leg muscle mass and walking speed in both groups, respectively. All statistical analyses were carried out using IBM SPSS version 25 (IBM Japan, Tokyo, Japan), with p < 0.05 considered statistically significant.

### Ethical review committee statement

Each author certifies that his or her institution approved the human protocol for this investigation and that all investigations were conducted in conformity with ethical principles of research (IRB#5-17-7, 5-19-20, 18-Io-158-2). All procedures performed in studies involving human participants were in accordance with the 1964 Helsinki declaration and its later amendments or comparable ethical standards.

## Results

Thirty-nine (87%) of the 45 female patients with DHS examined during the study period agreed to participate. A total of 23 patients met the exclusion criteria (Fig. 1), comprising of 17 patients with secondary DHS, 5 with gait disturbance, and 1 with metal in the body (total knee arthroplasty). After exclusions, 16 patients with idiopathic DHS were included in this study with an average time from onset of 31.1 ± 26.6 months. The mean age of the 32 healthy volunteers was 75.4 (range 63–89, 32 females). No one had a history of malignant tumor and diabetes in both groups. There was no missing data in this study.

### Prevalence of sarcopenia

There was no significant difference in age and height between the two groups, while the body weight and BMI in the DHS group were significantly lower than those in the healthy group (p < 0.001, p < 0.001) (Table 1). No patients in the DHS group had 3.0 g/dl ≥ serum albumin or 150 mg/dl ≥ total cholesterol. The average chin-brow vertical angle (CBVA) and C2-7 sagittal vertical axis (SVA) in the DHS group were 66.0 degrees (range 28–87) and 76.4 mm (range 49–97), respectively, and the heads of patients were markedly drooping. Three of 16 patients (18.8%) with idiopathic DHS had SMI < 5.7 kg/m^2^, one of the diagnostic criteria for sarcopenia. All three were diagnosed with sarcopenia due to their grip strength and/or lower walking speed compared to criteria values (Table 2). Of the 32 healthy subjects, 6 of 7 patients with SMI < 5.7 kg/m^2^ were diagnosed with sarcopenia. One patient had no sarcopenia because both grip strength and walking speed were higher than criteria values. The prevalence of sarcopenia was not significantly different between the two groups (p = 1.000).

**Table 1:** Demographic data of the participants.

	DHS group	Healthy group	p value
Age, years	75.4 ± 7.4 (64–88)	75.4 ± 7.3 (63–89)	1.000
Height, m	1.48 ± 0.08 (1.36–1.63)	1.50 ± 0.07 (1.36–1.65)	0.325
Mass, kg	43.4 ± 6.5 (31.3–53.4)	53.2 ± 9.3 (37.7–73.5)	** < 0.001**
BMI, kg/m^2^	19.7 ± 2.1 (15.5–25.0)	23.5 ± 3.7 (17.1–30.0)	** < 0.001**
Serum albumin, g/dl	4.1 ± 0.4 (3.3–4.9)	–	–
Total cholesterol, mg/dl	227.6 ± 33.0 (180.0–306.0)	–	–
Apex, cervical/thoracic	7/9	–	–
CBVA, degrees	66.0 ± 15.6 (28.0–87.0)	–	–
C2-7 angle, degrees*	− 35.0 ± 20.2 (− 75.0 to 8.0)	–	–
C2-7 SVA, mm	76.4 ± 12.4 (49.0–97.0)	–	–

Data are presented as the mean ± SD (range). Bold figures indicate statistical significance with *p* < 0.05.

*DHS* dropped head syndrome; *BMI* body mass index; *CBVA* chin–brow vertical angle; *SVA* sagittal vertical axis.

*Positive value indicates lordosis.

**Table 2:** Comparison of prevalence and clinical outcome between groups.

	DHS (n = 16)	Healthy (n = 32)	Mean diff	95% CI (lower)	95% CI (upper)	p value
Diagnosis of sarcopenia, number (%)	3 (18.8)	6 (18.8)	–	–	–	1.000
SMI, kg/m^2^	6.08 ± 0.44	6.17 ± 0.70	− 0.08	− 0.47	0.33	0.669
Grip strength, kg	18.4 ± 3.0	21.7 ± 5.0	− 3.3	− 6.1	− 0.6	**0.019**
Usual gait speed, m/s	0.8 ± 0.2	1.3 ± 0.3	− 0.5	− 0.6	− 0.3	** < 0.001**
Arm muscle mass, kg/m^2^	1.21 ± 0.12	1.37 ± 0.21	− 0.16	− 0.27	− 0.04	**0.008**
Leg muscle mass, kg/m^2^	4.89 ± 0.34	4.79 ± 0.57	0.10	− 0.21	0.41	0.526
Trunk muscle mass, kg/m^2^	7.92 ± 0.53	8.51 ± 0.71	− 0.59	− 1.00	− 0.19	**0.005**
FMI, kg/m^2^	4.73 ± 1.66	8.01 ± 2.87	− 3.28	− 4.85	− 1.72	** < 0.001**
FFMI, kg/m^2^	14.89 ± 0.69	15.52 ± 1.30	− 0.63	− 1.34	0.07	0.076
FFMI/FMI ratio	3.50 ± 1.17	2.19 ± 0.81	1.31	0.72	1.89	** < 0.001**

Data are presented as mean ± SD. Bold figures indicate statistical significance with *p* < 0.05.

*DHS* dropped head syndrome; *SMI* skeletal muscle mass index; *FMI* fat mass index; *FFMI* fat-free mass index.

### Trunk and appendicular muscle mass

**Figure 2:**
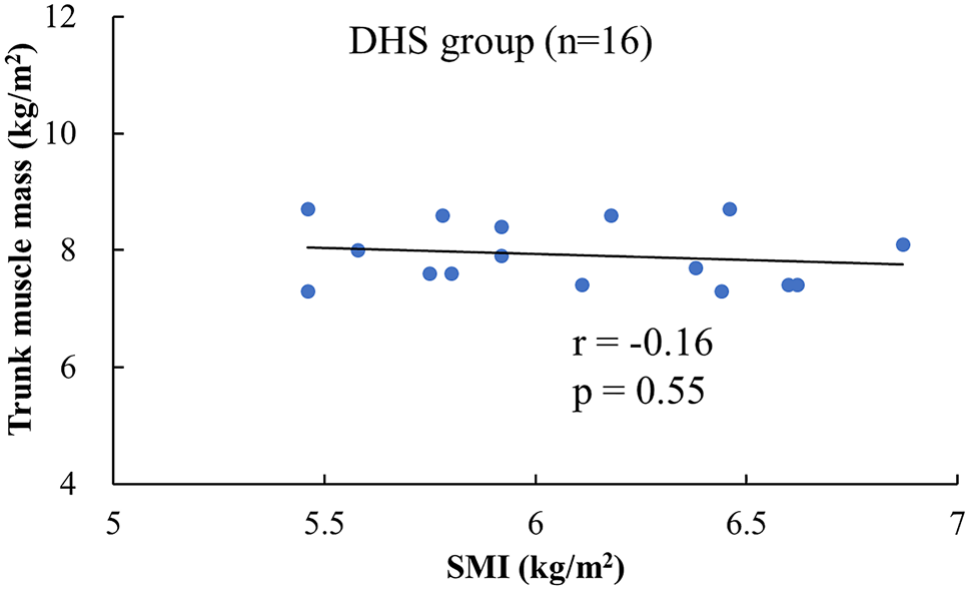
Correlation between SMI and trunk muscle mass in the DHS group. *DHS* Dropped head syndrome; *SMI* skeletal muscle mass index.

**Figure 3:**
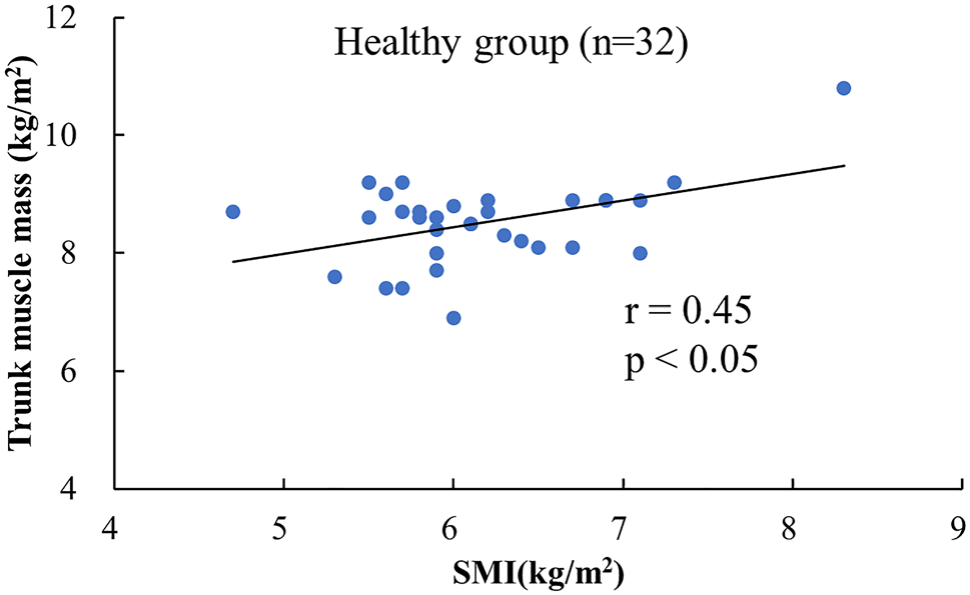
Correlation between SMI and trunk muscle mass in the healthy group. *DHS* Dropped head syndrome; *SMI* skeletal muscle mass index.

Trunk muscle mass and FMI in the DHS group were significantly lower than those in the healthy group: trunk muscle mass was 7.92 ± 0.53 kg/m^2^ in the DHS group versus 8.51 ± 0.71 kg/m^2^ in controls (mean difference − 0.59 [95% CI − 1.00 to − 0.19]; p = 0.005), and FMI was 4.73 ± 1.66 kg/m^2^ in the DHS group versus 8.01 ± 2.87 kg/m^2^ in controls (mean difference − 3.28 [95% CI − 4.85 to − 1.72]; p < 0.001). The FFMI-FMI ratio, which accounted for the physique, was significantly higher in the DHS group than in the control group (mean difference 1.31 [95% CI 0.72 to 1.89]; p < 0.001) (Table 2). No significant correlation was found between the SMI and trunk muscle mass in the DHS group, while a significant correlation was found between them in the healthy group (p < 0.05, r = 0.45) (Figs. 2, 3). No significant correlation was found between the leg muscle mass and walking speed in the DHS group, whereas a significant correlation was found in the healthy group (p < 0.05, r = 0.35).

## Discussion

The purpose of this study was to assess the prevalence of sarcopenia in patients with idiopathic DHS and whether loss of appendicular muscle mass is associated with loss of trunk muscle mass. Our data showed that sarcopenia was not associated with the onset of idiopathic DHS. This is the first report to accurately measure the prevalence of sarcopenia in DHS patients using the results of muscle strength and physical performance as well as muscle mass. The prevalence of sarcopenia in our control group showed a similar rate to the prevalence of the disease in the Asian population (5.5–25.7%)^15^, which support the validity of our findings. Considering the blood parameters including serum albumin and total cholesterol in DHS patients, they were never undernourished. Based on our results, the prevalence of sarcopenia in DHS patients was not different from that in healthy individuals, while grip strength and walking speed were lower than in healthy individuals of the same age.

The AWGS 2019 algorithm^15^ is the most commonly used diagnostic criteria in recent studies of sarcopenia in the Asian population. In this algorithm, the decreased SMI is a prerequisite for sarcopenia, and both the grip strength for muscle strength evaluation and the walking speed for physical function evaluation are used as diagnostic criteria. Although the DHS group exhibited decreased muscle strength and physical function, we believe the low 20% prevalence of sarcopenia in this study was due to the lack of decrease in SMI. Our prevalence rate is considerably different from a previous study by Eguchi et al.^17^ that reported 70%. Comparing the DHS patients between the two studies, there were no significant differences in race, height, weight, or BMI, and cervical malalignment. In 2010, The European Working Group on Sarcopenia in Older People (EWGSOP)^20^ defined elderly people with reduced SMI but without reduced walking speed or muscle strength as pre-sarcopenia. An algorithm was developed specifically for the Asian population in 2019, and pre-sarcopenia was redefined as non-sarcopenia. The prevalence of sarcopenia in patients with DHS in the previous study^17^ may be overdiagnosis, as it did not adopt the criteria of the AWGS 2019 algorithm^15^.

We found that the decreased trunk muscle mass (mean value: 7.92 ± 0.53 kg/m^2^) was characteristic of DHS patients compared to healthy subjects. There was a significant correlation between SMI and trunk muscle mass in healthy subjects (r = 0.45, p < 0.05), but not in DHS patients (p = 0.55). The results show that DHS patients had low trunk muscle mass, even in those with high SMI. The musculoskeletal characteristics of DHS patients revealed a remarkable decrease in trunk muscle mass without the decrease in appendicular muscle mass that is characteristic of sarcopenia. Decreased trunk muscle mass has been reported to be associated with pain, spinal malalignment, and quality of life in patients with spinal disorders^21^, and we believe that decreased trunk muscle mass is associated with the development of idiopathic DHS. We have previously reported that exercise interventions focused on the lower trunk and cervical muscles in patients with DHS yield good outcomes^22^. From our findings, a focus on trunk muscle mass could be an option for conservative treatment of DHS patients. Although walking speed is correlated significantly with leg muscle strength^23^, DHS patients did not demonstrate a significant correlation between leg muscle mass and walking speed. It is also interesting to note that the results of this study showed no significant difference in leg muscle mass between the healthy elderly and patients with DHS. It has been reported that maintaining the elevated position of the head is essential for optimizing input from the visual, vestibular, and somatosensory systems and maintaining systemic balance during exercise^24,25^. In DHS patients, dropped head or reduced trunk function may contribute to decreased walking ability, regardless of leg muscle strength. Strength exercises aimed at increasing muscle mass in the lower extremities may not be effective in improving the reduced walking ability in patients with DHS.

Reports by Kyle et al.^26^ and Bahadori et al.^27^ show standard values for FFMI and FMI in non-Japanese populations, and these values for the elderly Japanese were examined in a cohort study of 4,500 people^28^. An increasing FMI with aging represented by sarcopenic obesity is regarded as one of the problems in an aging society^29^. There are no reports of FFMI and FMI in patients with orthopedic diseases, including DHS. Patients with DHS exhibited reduced FMI as opposed to age-related changes. FMI of female patients with DHS obtained in this study (mean 4.7 ± 1.7 kg/m^2^) was remarkably lower than that of healthy subjects in the previous study (mean 7.4 ± 2.6 kg/m^2^)^28^. Furthermore, the FFMI-FMI ratio in the DHS group was significantly higher than that in control groups. Therefore, we believe that reduced body fat mass is an indicator of DHS. Adipose tissue, along with skeletal muscle, is depleted by various chronic inflammations^30^. Fat loss in patients with DHS was not accompanied by reduced appendicular muscle mass. Although it is unclear why our findings differ slightly from the general patterns of age-related changes in body composition, trunk muscle and fat mass loss may occur in advance of DHS. We believe that DHS patients should be treated by focusing not only on muscle but also on fat. Adipose tissue has a rich composition of immune cells, and cytokines are important regulators of lipolysis^31^. Further investigation into the association between body composition of DHS patients and inflammatory markers is warranted.

Our research has some limitations. Firstly, patients with idiopathic DHS only included those who visited a single institution; therefore, their external validity is low, and it is difficult to generalize the results of this study. A multicenter research is needed to consider regional characteristics. Secondly, there was an admission rate bias, because the DHS group only included patients who visited the hospital. The prevalence of sarcopenia in DHS might be underestimated. Thirdly, the BIA method was used to evaluate body composition in this study. The most reliable tool for assessing body composition is the dual energy X-ray absorptiometry (DXA) method. The BIA method has a slightly lower accuracy in evaluating muscle mass. However, the BIA method provides a portable and inexpensive means for assessment without radiation exposure, which was the most suitable and practical method when considering the ethical aspect of radiation exposure to the control group. In the future, the comparative validity of DXA and BIA should be verified. Moreover, using MRI to examine the fat in the muscles of the trunk and neck at each spine level may be useful information for future treatment. Finally, the prevalence of sarcopenia and fat mass may be underestimated in this study due to the extremely small sample size. Utilizing a larger sample may have revealed a statistically significant difference between patients and the control. In future research, verification should be carried out by increasing the number of people to be measured. However, since DHS is a rare disease, our findings valuable basic research data.

## Conclusions

This study evaluated muscle mass, strength, and physical performance in female patients with idiopathic DHS and investigated the prevalence of sarcopenia according to the criteria of the AWGS 2019 algorithm^15^. Subsequently, sarcopenia was not associated with the onset of idiopathic DHS. The prevalence of sarcopenia in patients with DHS was approximately 20%, which was similar to that of age- and gender-matched healthy subjects. Our findings suggested that a decreased trunk muscle and fat mass was associated with female patients with idiopathic DHS, rather than sarcopenia and/or SMI.
